# Electrophysiologic Biomarkers for Assessing Disease Progression and the Effect of Riluzole in SOD1 G93A ALS Mice

**DOI:** 10.1371/journal.pone.0065976

**Published:** 2013-06-06

**Authors:** Jia Li, Minhee Sung, Seward B. Rutkove

**Affiliations:** Department of Neurology, Harvard Medical School, Beth Israel Deaconess Medical Center, Boston, Massachusetts, United States of America; Inserm, France

## Abstract

**Objective:**

To compare electrical impedance myography (EIM) 50 kHz phase to weight, motor score, paw grip endurance (PGE), CMAP amplitude, and MUNE for the identification of disease progression and the effect of riluzole in the SOD1 G93A mouse.

**Methods:**

Twenty-three animals received 8 mg/kg/day riluzole in the drinking water starting at 6 weeks of age; 22 animals served as controls. Weight, motor score, PGE, CMAP, MUNE, and EIM were performed weekly to evaluate disease progression.

**Results:**

No difference in clinical disease onset or survival was found between treated and untreated groups. In addition, all methods failed to identify any beneficial effect of riluzole. Thus, data from all animals were combined for additional analyses. Of the 4 parameters, EIM phase showed the earliest change from baseline and the most linear decline throughout the entire measurement period. In addition, EIM phase correlated with PGE, CMAP amplitude, and MUNE (Spearman r = 0.92, 0.90, and 0.72, respectively, p<0.01 for all). The rate of EIM phase decline also correlated with individual animal survival (Spearman r = −0.31, p<0.05).

**Conclusions:**

At this dose, riluzole is ineffective in slowing progression of ALS. However, EIM phase shows early linear declines, supporting its potential as a useful new biomarker for preclinical drug testing.

## Introduction

Outcome measures such as survival or the ALS Functional Rating Scale-Revised (ALSFRS-R) are used to assess new potential drug therapies in ALS clinical trials [1]–[4]. However, such approaches have limited sensitivity to disease progression and may require an extended length of time or large numbers of individuals to find a treatment effect [5]. Accordingly, biomarkers to more effectively measure disease progression over shorter periods of time and with fewer patients may offer the prospect of helping speed Phase 2 clinical trials, ultimately aiding in the identification of new treatments.

While such tools are especially important in human clinical trials, similar needs also exist for preclinical studies [6], [7]. The ability to more rapidly and accurately detect a treatment effect in mice is important since it can speed that therapy's introduction into clinical studies. In addition, the expanding array of ALS animal models demands more efficient tools for identifying treatment effects. Currently, most preclinical mouse studies rely on measures such as animal weight, survival, and various behavioral measures such as the paw grip endurance test or motor score [7]. Whereas these measures are clearly useful, some are relatively insensitive (e.g., weight) and following survival means that at least one-half year is needed to complete any study, the exact length depending on the model being utilized. Motor unit number estimation (MUNE) has also been studied to a limited extent in mice [8], but is not used routinely by most investigators given that it requires considerable training and is time-consuming to perform.

One technique that appears to have promise as a biomarker both in human and animal ALS is electrical impedance myography (EIM) [9], [10]. In EIM, a weak, high-frequency electrical current is passed across a set of electrodes overlying a muscle of interest and the consequent surface voltages are measured [11]. These voltages provide a measure of muscle pathology including muscle atrophy and the presence of fat and connective tissue within the muscle. Human studies have shown that EIM 50 kHz phase value is very reproducible and very sensitive to decline in ALS [9], [12]; in addition, the rate of deterioration in ALS correlates to the length of survival, suggesting that EIM could potentially serve as a surrogate outcome measure [9]. Similarly, EIM applied to the SOD1 G93A rat model demonstrated great sensitivity to disease progression as well as a strong relationship between the rate of decline in EIM and the length of survival of the animals [10].

Given the need for improved biomarkers for ALS mouse research, we have recently developed a straightforward, rapid, and reproducible approach for performing EIM in the mouse hind limb [13]. In that study, only cross-sectional data was evaluated, comparing healthy to ALS animals. In this study, we evaluate longitudinally a group of ALS SOD1 G93A mice treated with riluzole and a group of untreated animals to determine the ability of EIM to detect the rate of progression and whether a treatment effect of riluzole could be identified.

## Materials and Methods

### Animals

All studies were approved by the Beth Israel Deaconess Medical Center Institutional Animal Care and Use Committee (IACUC). Breeding colonies of ALS SOD1 G93A (strain: B6SJL-Tg(SOD1-G93A)1Gur/J) mice were established from animals obtained from Jackson Laboratories (Bar Harbor, Maine). Animals were genotyped by tail snip and were weaned at 21 days. A total of 47 ALS SOD1 G93A mice were studied (23 females and 24 males) and fed a standard diet *ad libitum*. Animals were monitored on a daily basis by research staff, evaluating condition of fur, grooming, and the presence of porphyrin staining. Animals were given gel-packs (DietGel® 76A, PharmaSer, Framingham, MA) when their limbs became too weak to reach food. They were sacrificed with an intraperitoneal dose of Fatal Plus® (Patterson Veterinary Supply, Inc., Devens, MA) at the point that their hind limbs became completely paralyzed and they could no longer successfully reach the gel-pack for feeding. Unfortunately, our IACUC would not approve the standard approach of waiting for the animal to right itself after 30 seconds of being placed on its side, and thus this alternative approach was used for all animals.

Animals were evenly divided into riluzole-treated and untreated groups and weights were obtained weekly. Our goal was to perform a high-quality controlled drug study taking into the account the issues discussed in Scott et al [14].

### Treatment

Riluzole (Sigma-Aldrich) was dissolved in the animals' drinking water, aiming for a total dose of 8 mg/kg/day based on the study by Kennel et al [15]. Although this dose was lower than that used in two other studies of mouse motor neuron disease [16], [17], it is the only dose that demonstrated an actual slowing of disease onset and a prolongation of survival. Moreover, it was the only dose that was not found to be ineffective in later studies [14]. Treatment was initiated at approximately 6 weeks of age in all animals.

### Behavioral measurements

A standard motor score assessment was performed to determine clinical disease onset [7]. A score of 4 was given for animals with no sign of motor dysfunction; 3 for animals with tremors when suspended by the tail; 2 when the animals had mild difficulty ambulating; 1 when they were dragging at least one of their hind limbs; 0 when both hind limbs were fully paralyzed. The paw grip endurance test was also performed as previously described [7].

### MUNE measurements

Motor unit number estimation (MUNE) was performed using a TECA Synergy T2 EMG Monitor System (Viasys, Madison, WI) on the left hind limb stimulating the sciatic nerve at the sciatic notch and recording via disposable ring electrodes around the entire distal leg, with a ground electrode placed on the right hind paw, as previously described [8]. All MUNE was performed by a single individual (JL) using the standard incremental method [8]. Briefly, a supramaximal compound motor action potential (CMAP) was obtained; then, the stimulus intensity was reset to zero and gradually increased until the first motor unit potential (MUP) was identified. Stimulus intensity was further increased until a total of 20 such steps in amplitude were obtained. From this data, the average single motor unit potential amplitude was obtained. The CMAP amplitude was divided by this value to obtain the MUNE.

### Animal EIM measurements

All EIM measurements were performed with the animals placed under 1% isoflurane anesthesia delivered through a nose cone with a heating pad underneath the limb to maintain consistent temperature, as previously described [13]. After the fur was clipped, a depilatory agent was applied to the skin to remove all remaining fur; the skin was cleaned with 0.9% saline solution. The leg was then taped to the measuring surface at an approximately 45° angle extending out from the body, away from the head. A pinpoint tattoo was placed close to the center of the gastrocnemius muscle at a point approximately 2/3 of the distance between the midpoint of the lumbar spine and the base of the heel pad of each mouse in order to ensure consistent electrode placement over subsequent measurement sessions.

A fixed rigid 4-electrode array was applied over the gastrocnemius muscle [13]. EIM measurements were performed with the Imp SFB7® bioimpedance spectroscopy device (Impedimed, San Diego, CA), which obtains impedance data from 3 kHz to 1 MHz. However, in this analysis we only utilized the single frequency 50 kHz data.

### Data analysis

All data were summarized as mean ± standard error across the groups. Kaplan-Meier curves were constructed to assess the effect of riluzole on the length of survival and clinical time of disease onset (as based on the motor score). Unpaired t-tests were used to compare the means between 2 groups; Spearman's correlation coefficient was calculated to assess the relationship between survival and rate of decline in MUNE and EIM measurements as well as for correlations between EIM phase and PGE, CMAP amplitude and MUNE. For nominal data (e.g., motor score normal versus abnormal), Fisher exact test was utilized. For all analyses, significance was accepted at p<0.05, two-tailed.

## Results

Two of the 47 animals were excluded from the analysis because they died at young ages for reasons unrelated to ALS. One animal escaped and was fatally injured during its retrieval. The other developed a periorbital infection and the animal research facility requested its euthanization. Thus, a total of 23 treated and 22 untreated animals were used in the following analyses.

### Effect of riluzole

**Figure 1:**
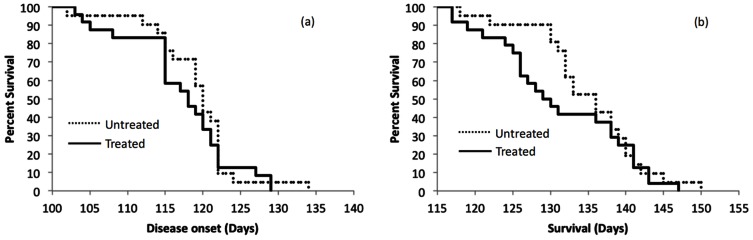
Kaplan-Meier Curves showing survival and disease onset based on standard clinical measures. a. Disease onset, b. Survival.

Figure 1 shows Kaplan-Meier curves for both the untreated and treated animals. The mean clinical disease onset for untreated animals was no different from the treated animals (120.1±1.3 days versus 118.3±1.4, respectively, p = 0.35, Figure 1a). The mean survival for the untreated group was not different from that of the treated group (136.1±1.6 days versus 132.4±1.8 days, respectively, p = 0.13, Figure 1b). Based on the standard deviations of the group survival values and the number of animals in each group, there had been approximately an 86% power to detect a 7.5 day prolongation in survival (p<0.05, one-tailed).

**Figure 2:**
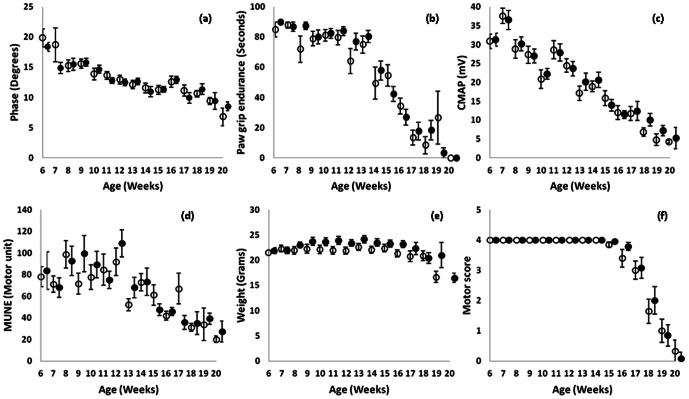
Weekly measures for riluzole and non-riluzole treated animals. a. 50 kHz phase, b. Paw grip endurance, c. CMAP amplitude, d. MUNE, e. weight, f. motor score. Open circles, untreated; closed circles, treated.

As shown is Figure 2a–f, none of the rates of decline for EIM, PGE, CMAP amplitude, MUNE, weight or motor score showed significant difference between untreated and treated animals, from 6 weeks of age until death, although there was a non-significant difference in weight (Figure 2e). This data is also summarized in Table 1, which shows the individual slopes of decline over time for each parameter (including EIM resistance and reactance) for riluzole-treated and untreated animals.

**Table 1:** Rates of decline from 6 weeks onward for EIM, PGE, CMAP amplitude, and MUNE in untreated, treated animals and combined (treated+untreated) groups.

	Untreated	Treated	p value*	Combined
**Resistance (Ω/Week)**	0.13±1.63	−2.22±1.06	0.23	−1.12±0.95
**Reactance (Ω/Week)**	3.19±0.68	2.53±0.53	0.45	2.84±0.42
**Phase (°/Week)**	0.62±0.13	0.56±0.08	0.67	0.59±0.07
**Paw grip endurance (sec/Week)**	6.78±0.59	8.32±0.65	0.09	7.10±0.41
**CMAP (mV/Week)**	2.25±0.20	2.17±0.17	0.76	2.21±0.13
**MUNE (Motor unit/Week)**	3.38±0.91	3.68±1.23	0.84	3.54±0.77
**Weight (milligrams/week)**	27.4±50.4	−2.0±50.9	0.72	11.3±39.7
**Motor score (points/week)**	0.189±0.08	0.189±0.08	0.97	0.189±0.08

* p values compare rates of decline for treated and untreated groups.

### Alterations in parameters over time

Since the riluzole- treated animals did not show a significant difference from untreated animals in any of the parameters, the treated and untreated animal data were combined for all the following analyses. The combined rates of decline are also summarized in Table 1.

**Figure 3:**
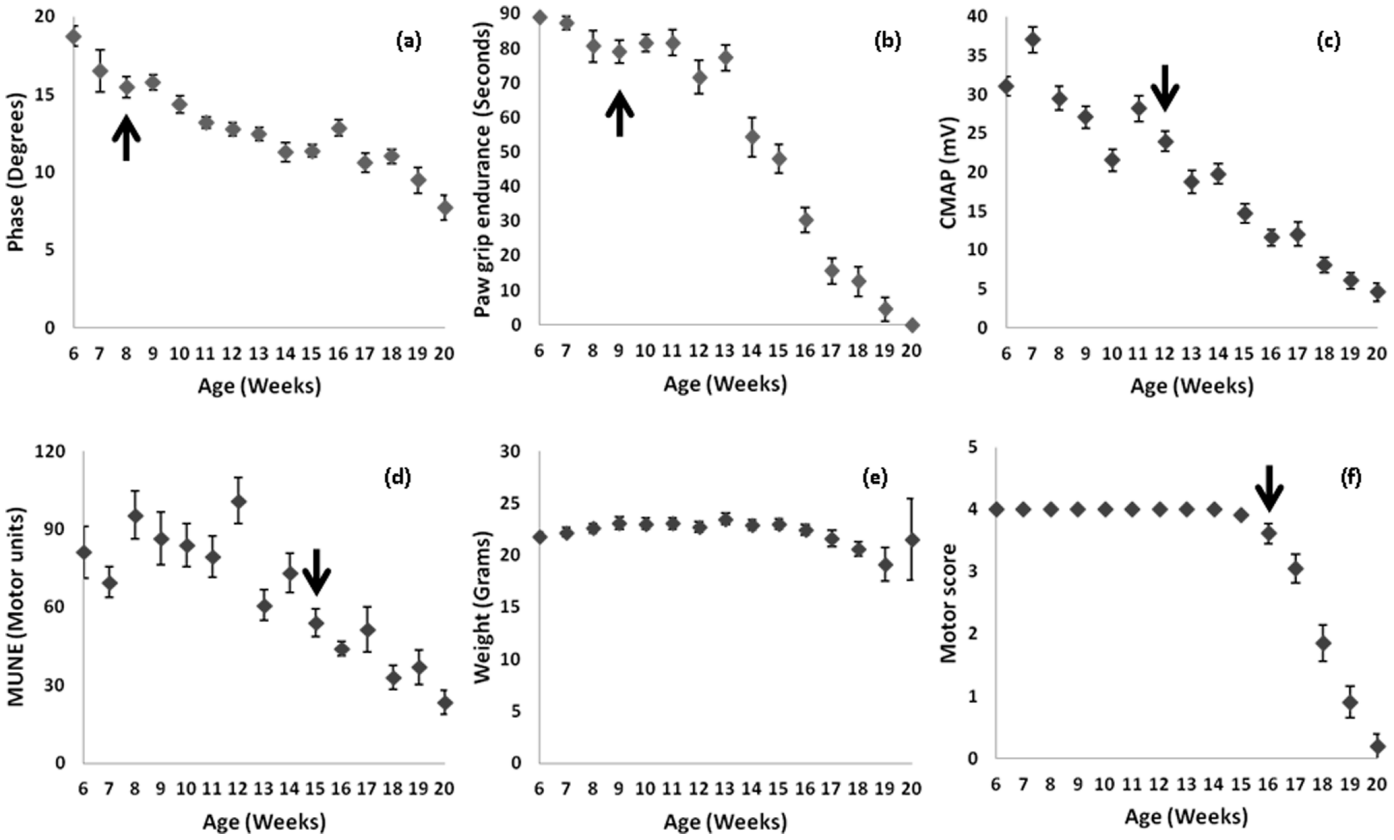
Combined riluzole and non-riluzole treated animals. a. 50 kHz phase, b. Paw grip endurance, c. CMAP amplitude, d. MUNE, e. weight, f. motor score. Arrows indicate time of first consistent significant difference from baseline values for that measure.

Figure 3a–f provides a comparison of EIM phase, PGE, CMAP, MUNE, weight and motor score over time with data from both groups combined. The arrows depict the time of significant persistent change from baseline (p<0.05) for two consecutive weeks or longer; this information is also provided in Table 2. Of note, 50 kHz phase showed a considerably earlier change from baseline (at 8 weeks) than did CMAP or MUNE, although only modestly better than PGE (9 weeks). Weight, previously a commonly used measure, did not show a significant change from baseline at any point, making it a similarly insensitive parameter for disease onset. (A decline relative to maximum weight, however, was present at 18 weeks.) Likewise, the motor score did not show a significant change until 16 weeks.

**Table 2:** Linearity of progression and time until first consistent significant difference from baseline for the entire cohort of animals (N = 45).

Measure	Normalized mean absolute residual from fitted line†	Time of disease onset (weeks)
**Resistance** *	-	-
**Reactance**	0.06	8
**Phase**	0.06	8
**Paw grip endurance**	0.17	9
**CMAP amplitude**	0.10	12
**MUNE**	0.15	15
**Weight** *	-	-
**Motor score**	0.20	16

* Resistance and weight values did not change significantly from baseline;

† Residuals from fitted line normalized to mean value of that parameter across all weeks of measurement.

Table 2 also summarizes the linearity of the decline and the time of disease onset over the entire period of measurement for each of the markers. The linearity of decline was quantified by performing a least-squares fit based on the average data for each week across the entire measurement period. Then, residuals for each of the weeks were calculated; the absolute value of these were taken and averaged for each measure and then divide for the average value of that parameter across all measurement points, thus providing a gauge of how each week's data deviated from the regression line. As can be seen, EIM phase has by far the smallest mean residual, putting in numerical terms what is visually apparent in Figure 3: that EIM data would track very closely along a fitted line. Thus, although PGE showed a significant change from baseline at just 9 weeks, its behavior is decidedly non-linear (which is also visually apparent in Figure 3b.) Even compared to CMAP, which also has a fairly low average residual, the EIM residuals were significantly lower (p<0.001); moreover, CMAP did not become significantly different from baseline until 12 weeks of age.

### Correlations between survival and rate of deterioration

**Figure 4:**
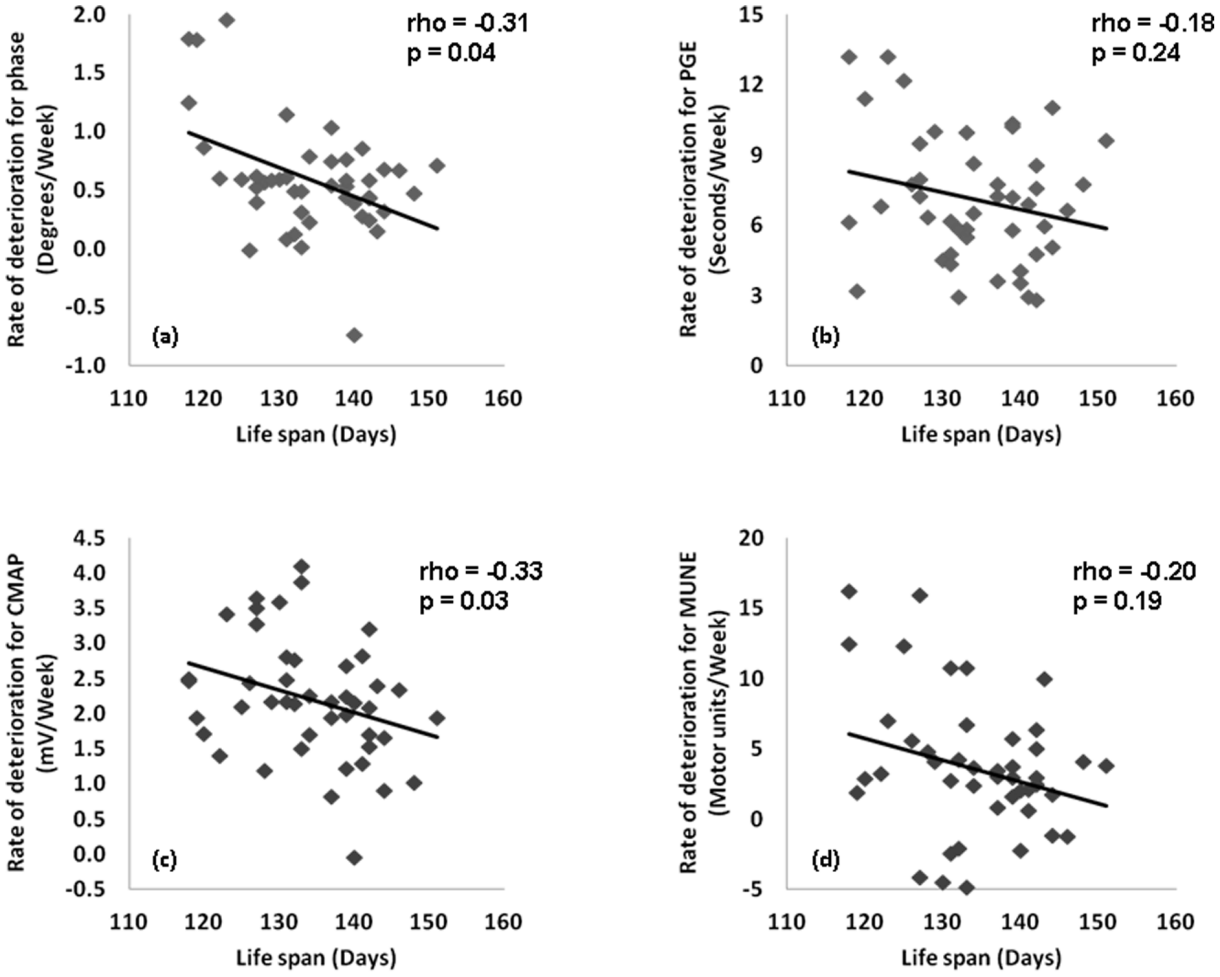
Correlation of rate of deterioration from 6 weeks of age until death with: a. 50 kHz phase, b. Paw grip endurance, c. CMAP amplitude, d. MUNE.

Figure 4a–d shows the correlation between the mean rate of deterioration from 6 to 20 weeks for EIM, CMAP, MUNE, and PGE with survival for that animal. Both EIM phase and CMAP amplitude fared similarly with correlation coefficients of R = −0.31 and R = −0.33, respectively, p<0.05 for both. Meanwhile, neither the rate of PGE or MUNE deterioration demonstrated significant correlations. It is important to recall, however, that the variation in lifespan among the animals is fairly small and thus demonstrating correlations between the rate of progression and survival is challenging.

### Correlations between EIM phase and other measures

**Figure 5:**
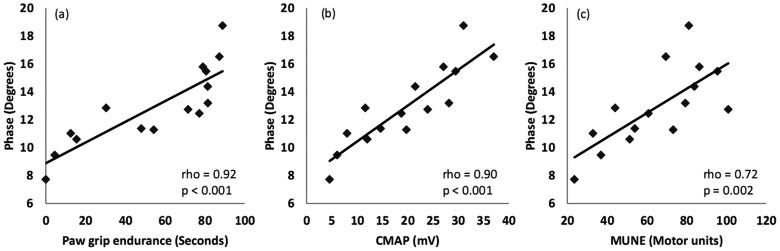
Correlation plots of EIM 50 kHz phase with PGE (a), CMAP amplitude (b) and MUNE (c). For this analysis, weekly data from all 45 animals is averaged.

Figure 5a–c shows the correlations between EIM 50 kHz phase and PGE, CMAP amplitude, and MUNE. For this analysis, the data from each week was averaged across all the animals. As can be seen, EIM correlates strongly with all three measures.

## Discussion

These results indicate that despite evaluating a number of parameters, riluzole at an approximate dose of 8 mg/kg/day has no beneficial effect in SOD1 G93A mice. The complete absence of an effect of riluzole in these studies may at first seem puzzling, since earlier studies reported a modest therapeutic effect of riluzole in ALS mouse models [15]–[17]. However, more recent work has indicated the considerable limitations of those studies, and repeat testing, correcting for those limitations, showed that riluzole was ineffective at a variety of doses [14]. Our work appears to support that conclusion.

As can be seen in Figure 3, all the biomarkers are able to follow disease progression in this ALS mouse model, although weight and motor score appear especially insensitive to disease status. Of all the biomarkers, EIM appears to be perhaps the strongest for several reasons. First, EIM is able to identify a decline in the muscle at just eight weeks of age, earlier than any of the other measures. Second, the decline is remarkably linear with very little variability over time, thus making it possible to follow disease over short periods to determine a rate of progression rather than the lifespan of an animal. Thus, while PGE endurance shows a significant difference from baseline just one week later than EIM, it is very non-linear in the early weeks (Figure 3). CMAP, while being reasonably linear, does not show a significant change from baseline until 4 weeks later than EIM. Finally, the rate of decline of EIM, as well as of CMAP amplitude, but not in MUNE or PGE, appears to correlate to survival of the animals, thus implying that the rate of decline in the measure is predictive of an animal's eventual time of death.

By correlating EIM to PGE, MUNE and to CMAP amplitude, we aimed to explore the relevance of the EIM data to these different parameters. As these analyses reveal, there is a good correlation with all three measures, supporting that the alterations EIM detects reflect motor neuron loss, subsequent muscle fiber atrophy and declining motor function. The major change in EIM phase is due to reductions in reactance values as the disease progresses (as suggested by Tables 1 and 2) because resistance barely alters in these animals over time. This likely reflects the effects of atrophy with minimal change in the actual composition of the muscles [11]. Moreover, recent work has also demonstrated that the current flow in EIM is mainly restricted to the more superficial regions of the muscle [18]. Thus, we anticipate that EIM is most sensitive to alterations in the upper layers of the gastrocnemius muscle, a region that consists of mainly Type 2 fibers. Indeed, motor neurons innervating Type 2 fibers are more likely to be vulnerable early in the disease. It may be partially for this reason that EIM shows such sensitivity to change early in the disease course [19].

Another interesting observation from this study is the potential of CMAP amplitude to be a better biomarker for disease progression than MUNE. MUNE has been the focus of most electrophysiologic studies in the motor neuron disease realm for a number of years since it is conceptually appealing, as it offers a true estimation of the number of motor neurons innervating a muscle or group of muscles. However, despite improved approaches for its application, it continues to be more challenging to perform and interpret, thus requiring far more operator training, operator decision-making, and data collection time than either CMAP or EIM. From our data here, it also appears to be relatively insensitive to change early in disease. In humans too, the estimate becomes most consistent in advanced disease when relatively few motor units remain [20]. CMAP amplitude has recently been proposed as an alternative biomarker in spinal muscular atrophy [21] and thus re-evaluating its use as a primary marker of decline in ALS is warranted. In fact, early studies showed similar consistent reductions in the CMAP amplitude [22].

The limitations to this study include the fact that it was not blinded to the presence or absence of the drug; however, this issue would probably only be of concern if a treatment effect had been identified. Second, we studied only a single dose of riluzole that had previously been shown to be effective [15]. Ideally, it would have been preferable to study several doses; however, recent work has shown that higher dose riluzole has no therapeutic impact in these animals [14]. Moreover, there is the possibility that any drug, including riluzole, could display hormetic behavior (an upside-down U-shaped dose-response curve) [23], [24]. In such a situation, a higher dose may actually be less effective than a lower. Another limitation of the study is that the interpretation of the validity of biomarkers using this animal model is challenging since it is difficult to show that the biomarker correlates to survival because the animals die over a very short duration of time. Still, significant correlations were identified, consistent with results found for SOD1 G93A rats [10]. A final limitation of the study is the lack of any standardized EIM measurement tool for mice. Thus, in order to replicate these findings, it will be necessary to develop an array with an identical footprint to the one used here. However, EIM measurement tools continue to be developed, and it is expected that a standardized version for mouse work will be available soon.

Another general limitation of this work is the focus of studying biomarkers individually rather than combining values to produce stronger predictors of disease progression. Indeed, recent work by others has suggested that such composite markers could be more effective than individual ones in terms of both establishing a rate of progression and in its predictive abilities. Such work has been pursued in Alzheimer's disease [25] and multiple sclerosis [26]. To do so, however, would require larger sample sizes than those presented here, such that one set of data could be used to develop the composite marker and a second sample could be used to test it, so as to avoid “overfitting” the data. This should remain a goal of future research in this area.

Based on these results, one additional interesting observation that deserves closer study is how EIM values change in the early weeks of observation. It is clear that ALS SOD1 animals are different from healthy animals from a young age; for example, changes in motor neuron excitability have been demonstrated as early as the second post-natal week [27]. The fact that EIM values start to decline consistently during these early weeks may be particularly valuable since it could provide a very fast and efficient means of assessing the efficacy of a new therapy. Proof of this potential use, however, will require further study, and most challenging, a truly effective therapy.
